# Toll-Like Receptor 4 as a Favorable Prognostic Marker in Bladder Cancer: A Multi-Omics Analysis

**DOI:** 10.3389/fcell.2021.651560

**Published:** 2021-06-01

**Authors:** Jun-Lin Lu, Qi-Dong Xia, Yi Sun, Yang Xun, Heng-Long Hu, Chen-Qian Liu, Jian-Xuan Sun, Jin-Zhou Xu, Jia Hu, Shao-Gang Wang

**Affiliations:** Department of Urology, Tongji Hospital, Tongji Medical College, Huazhong University of Science and Technology, Wuhan, China

**Keywords:** toll-like receptor, bladder cancer, bioinformatics, prognosis, innate immunity, methylation

## Abstract

**Background:**

The toll-like receptor 4 (TLR4) agonist, Bacille Calmette-Guérin, has exhibited gratifying effects in treating bladder cancer. The study aims to explore the expression pattern, prognostic value, and potential mechanism of TLR4 in bladder cancer.

**Methods:**

The transcriptome file from the GSE13507 dataset in the Gene Expression Omnibus database and the promoter methylation file from the bladder cancer dataset in The Cancer Genome Atlas database were downloaded for analysis. The prognostic value of the TLRs was assessed by univariate Cox regression. Immunohistochemistry was applied to verify the expression of TLR4 in bladder cancer. The drug response is estimated through the R package “pRRophetic.” The CIBERSORT algorithm was carried out to estimate the infiltrating immune cells of samples. Gene Set Enrichment Analysis (GSEA) was performed to identify the pathways involved under varied TLR4 expression levels.

**Results:**

TLR4 is decreased in tumor tissues compared with surrounding tumor tissues or normal tissue, which is also positively correlated to the overall survival rate (hazard ratio [HR] = 0.38) and cancer-specific survival rate (HR = 0.15) of patients with bladder cancer. Low expression of TLR4 is observed in tumors with malignant performance (high pathological grade, higher tumor stage, and progression). Patients with low TLR4 levels are more sensitive to gemcitabine rather than cisplatin. The promoter methylation level of TLR4 is positively associated with TLR4 expression (*P* < 0.001). The cg14629571 methylation site largely contributes to the overall methylation level. The CIBERSORT analysis shows that high TLR4 expression is associated with lower levels of plasma cells, M0 macrophages, and M1 macrophages. The GSEA results indicate that the TGF-β pathway and apoptosis are activated in high TLR4 bladder cancer, while G2M checkpoint and E2F targets pathways are enriched in low TLR4 bladder cancer.

**Conclusion:**

This research discusses the abnormal expression and prognostic value of TLR4 in bladder cancer. The TLR4 expression can effectively predict oncological outcomes and drug sensitivity of bladder cancer patients. TLR4 is also associated with infiltrating immune cell variation and cancer pathway dysregulation. The results provide a novel prognostic marker and potential drug targets for bladder cancer.

## Introduction

Toll-like receptors (TLRs), including TLR1 to TLR10, belong to a class of highly conserved molecules. TLRs are mainly expressed in immune cells to recognize pathogen-associated molecular patterns or damage-associated molecular patterns (Kawasaki and Kawai, 2014). Recent studies also reveal the antitumor effects of TLRs (Ayala-Cuellar et al., 2019). Innate immune cells can secrete cytokines to kill cancer cells, or recruit adaptive immune cells through the activation of TLR signaling pathways (Olbert et al., 2015a; Dajon et al., 2017). The TLR agonists can promote apoptosis, inhibit proliferation, and restrain migration by directly targeting TLRs on tumor cells (Han et al., 2016; Hsu et al., 2017). Certain TLR agonists have been applied in clinical practice. A typical example is the utilization of Bacille Calmette-Guérin (BCG), a TLR2, TLR4, and TLR9 triagonist, in preventing recurrence of non-muscle invasive bladder cancer (NMIBC; Babjuk et al., 2020).

Bladder cancer is the seventh most prevalent cancer in man and the eleventh most prevalent in both sexes (Ferlay et al., 2012). Approximately a quarter of patients present muscle invasive tumor at the initial diagnosis. They are recommended to undergo radical cystectomy (Babjuk et al., 2020). However, replacement of the bladder considerably affects the quality of life due to urinary dysfunction (Yang et al., 2016). On the other hand, a proportion of 31 to 78% NMIBC patients will relapse despite undergoing tumor resection (Sylvester et al., 2006). The application of BCG, a live-attenuated microbial agent, opens a new era of tumor therapy, which can significantly reduce the recurrence rate after regular intravesical instillation (Lamm et al., 1991). The efficacy of BCG is greater than other intravesical chemotherapy drugs (Zhuo et al., 2016; Wu et al., 2017). However, about thirty percent of patients will still progress to muscle invasive bladder cancer (MIBC) and receive radical cystectomy.

Although the TLR agonist has achieved remarkable results in bladder cancer treatment, little is known about the expression pattern and prognostic value of TLRs in bladder cancer. The mechanism responsible for the effect of TLRs on the tumor immune microenvironment remains unclear. The aim of this study is to evaluate the expression profile of TLR genes, especially TLR4, in bladder cancer. We conduct further analysis to indicate the changes of the promoter methylation levels of TLR4 and to reveal infiltrating immune cell changes and alteration of functional pathways.

## Materials and Methods

### Data Sources

Transcriptome and clinical information with survival data of the GSE13507 array were downloaded from the Gene Expression Omnibus database^1^. The promoter methylation data of bladder cancer from The Cancer Genome Atlas (TCGA) was obtained from the UCSC-Xena database^2^. The sequence of the promoter region of TLR4 was obtained from the NCBI (United States National Center for Biotechnology Information) database. We acquired the methylation sites of the TLR4 promoter according to the annotation of the Illumina Human Methylation 450 k platform.

### Expression of TLRs in Bladder Cancer

The GSE13507 array contained normal samples, surrounding tumor samples, and tumor samples. We compared the expression status of TLRs in the three tissue types. Then we evaluated the prognostic value of TLRs using overall survival (OS) and cancer specific survival (CSS). The patients are divided into a high TLR4 expression group and a low TLR4 expression group based on the median value of TLR4 expression. The Kaplan-Meier survival curves for OS and CSS were plotted for TLR4.

### Clinical Correlation and Immunohistochemistry Validation

Cisplatin and gemcitabine are the first line chemotherapy for muscle invasive bladder cancer. The drug response was calculated with the R package “pRRophetic” to predict each patient’s drug sensitivity of cisplatin and gemcitabine. We explored the correlation between the expression of TLR4 and patient/tumor characteristics in the GSE13507 array, including age, gender, pathological grade, tumor stage, regional lymph node metastasis, distant metastasis, progression status, and drug sensitivity. In addition, to verify the differential expression status of TLR4 between high-grade and low-grade or muscle-invasive and NMIBC tissues, we applied immunohistochemistry (IHC) to stain TLR4 in our cohort. The patient cohort was recruited under the approval of the Institutional Ethics Committee (ID: TJ-IRB20200729). The IHC was performed according to the manufacturer’s instructions. Briefly, the formalin-fixed, paraffin-embedded (FFPE) slices were deparaffinized with xylene and alcohol of gradient concentrations. The antigen retrieval procedure was performed using 0.01 M citrate buffer pH6.0. Endogenous peroxidase activity was blocked by incubation with hydrogen peroxide for 15 min. Thereafter, overnight incubation with primary antibody (TLR4: rabbit, 1:200, ab22048, Abcam) was carried out at 4°C. Secondary antibody incubation and diaminobenzidine staining were conducted using GTVisonTM + Detection System/Mo&Rb (GeneTech, Shanghai, China). To evaluate the expression of TLR4, the mean optical density (MOD) was calculated by averaging five representative fields of 400 × magnification. The staining in the tumor area (not stroma area) is included in quantification. The MOD of each field was analyzed by Image-Pro Plus 6.0 (Media Cybernetics, Inc.) software.

### Promoter Methylation of TLR4

The promoter methylation of TLR4 was analyzed using the methylation profile from TCGA BLCA. We acquired the methylation sites of the TLR4 gene promoter according to the annotation of the Illumina Human Methylation 450 k platform. After extracting the beta value of these sites in bladder cancer tissues, we explored the correlation between the expression of TLR4 and overall promoter methylation level (calculated by averaging the methylation beta-value of each site) as well as the promoter methylation level of each site. Finally, we investigated the correlation between the methylation level and patient/tumor characteristics.

### CIBERSORT and Gene Set Enrichment Analysis

To further explore the influence of TLR4 in immune microenvironment reprogramming, we estimated the infiltrating immune cells for each sample in GSE13507 using CIBERSORT algorithm. A *P* value was generated for each sample. The estimation was considered as a reliable result when *P* < 0.05. Then we compared the differential immune cell infiltration status using reliable samples between high and low TLR4 expression groups. Moreover, to determine the involving pathways, we employed the Gene Set Enrichment Analysis (GSEA) method to figure out the differentially activated pathways in the high or low TLR4 expression group. The HALLMARK and KEGG gene sets from MSigDB^3^ were utilized (Liberzon et al., 2015). In addition, we also assessed the relationship between TLR4 expression and cytokine related pathways using BIOCARTA gene sets.

### Statistical Analysis

The data were processed using the Strawberry PERL programming language (Version 5.30.2.1). All statistical analyses were performed using R software (version 4.0.2). Statistical difference between two continuous variables was assessed with the Mann–Whitney test. The Kruskal–Wallis test was used to detect the difference between multiple continuous variables. The log-rank test was applied to detect the difference between two survival curves. We used Cox regression analysis to assess the prognostic value of TLRs. The correlation between two continuous variables was indicated by the Pearson correlation coefficient. A *P* value < 0.05 was considered statistically significant. The symbols “^∗^”, “^∗∗^”, and “^∗∗∗^” refer to *P* values < 0.05, <0.01, and <0.001, respectively.

## Results

### Differential Expression Pattern and Prognostic Value of TLRs in GSE13507

**FIGURE 1:**
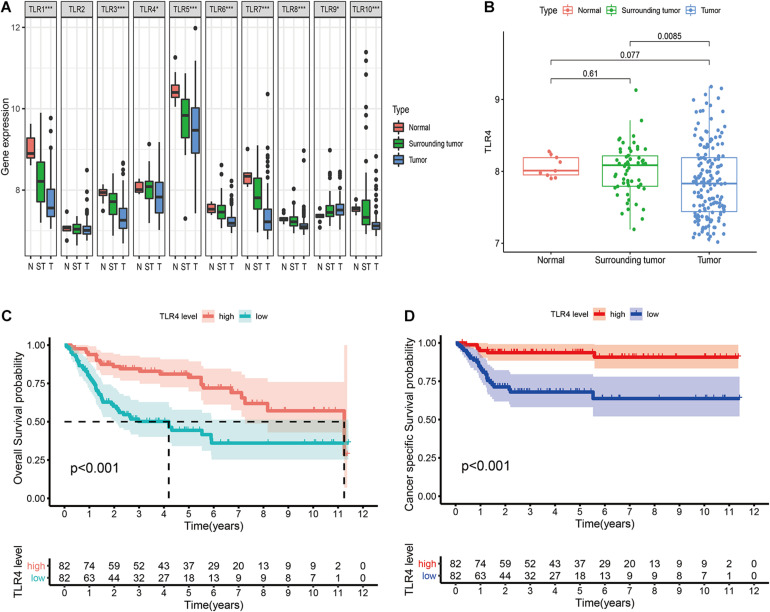
Comprehensive analysis of ten TLRs in bladder cancer in GSE13507. **(A)** Differential expression of 10 TLRs in normal tissue (*n* = 9), surrounding tumor tissue (*n* = 58), and tumor tissue (*n* = 165). **(B)** The TLR4 expression in three tissue types. **(C)** Prognostic value of TLR4 to predict the overall survival in bladder cancer patients. **(D)** Prognostic value of TLR4 to predict the cancer-specific survival in bladder cancer patients.

The GSE13507 array includes 9 normal tissues (one mouse bladder sample is excluded), 58 surrounding tumor tissues, and 164 primary tumor tissues (one human sample is excluded due to incomplete clinical information). The clinicopathological characteristics of the patients are shown in Table 1. The differential expression of TLRs between the three tissue types is illustrated in Figure 1A. The expression of TLR1, TLR3, TLR5, TLR6, TLR7, TLR8, and TLR10 show significantly decreasing trends from normal tissues to surrounding tumor tissues and to tumor tissues. However, TLR9 expression shows a reverse trend. As for TLR4, the expression is also reduced in tumor tissue compared to normal tissue (*P* = 0.077) and surrounding tumor tissue (*P* = 0.0085; Figure 1B). However, only TLR4 can effectively predict OS (HR = 0.38 [0.23–0.64], *P* < 0.001) and CSS (HR = 0.15 [0.06–0.39], *P* < 0.001) of bladder cancer patients (Table 2). The Kaplan-Meier curves also verify the prognostic value of TLR4 (Figures 1C,D).

**TABLE 1:** Clinicopathological characteristics of patients included in this study (GSE13507).

**Variables**	**Value**
Total patients	164
Male, *n* (%)	134 (81.7)
**Age, *n* (%)**	
>65 year	90 (54.9)
≤65 year	74 (45.1)
**Muscle invasiveness, *n* (%)**	
Non-muscle invasive	103 (62.8)
Muscle invasive	61 (37.2)
**Grade, *n* (%)**	
High grade	59 (36.0)
Low grade	105 (64.0)
Progression, *n* (%)	30 (18.3)
**Tumor stage, *n* (%)**	
Ta	24 (14.6)
T1	80 (48.8)
T2	31 (18.9)
T3	18 (11.0)
T4	11 (6.7)
**Regional lymph node metastasis, *n* (%)**	
N0	151 (92.1)
N1–3	13 (7.9)
**Distant metastasis, *n* (%)**	
M0	157 (95.7)
M1	7 (4.3)

**TABLE 2:** Univariate cox regression analysis of TLRs for both overall survival and cancer specific survival.

**Gene**	**Overall survival**		**Cancer specific survival**	
	**HR**	***P*-value**	**HR**	***P*-value**
TLR1	0.96 [0.58 to 1.59]	0.872	1.18 [0.60 to 2.31]	0.635
TLR2	0.43 [0.11 to 1.66]	0.220	0.43 [0.06 to 3.41]	0.428
TLR3	0.62 [0.32 to 1.22]	0.167	0.49 [0.17 to 1.35]	0.167
TLR4	0.38 [0.23 to 0.64]	<0.001	0.15 [0.06 to 0.39]	<0.001
TLR5	1.17 [0.88 to 1.57]	0.274	0.99 [0.65 to 1.50]	0.946
TLR6	0.80 [0.23 to 2.81]	0.730	1.43 [0.29 to 7.00]	0.657
TLR7	0.75 [0.43 to 1.32]	0.323	0.96 [0.47 to 1.99]	0.916
TLR8	0.78 [0.18 to 3.41]	0.745	0.91 [0.14 to 5.91]	0.924
TLR9	1.64 [0.64 to 4.19]	0.298	4.44 [1.44 to 13.69]	0.009
TLR10	2.22 [1.04 to 4.74]	0.039	1.91 [0.68 to 5.38]	0.220

### Clinical Correlation of TLR4

**FIGURE 2:**
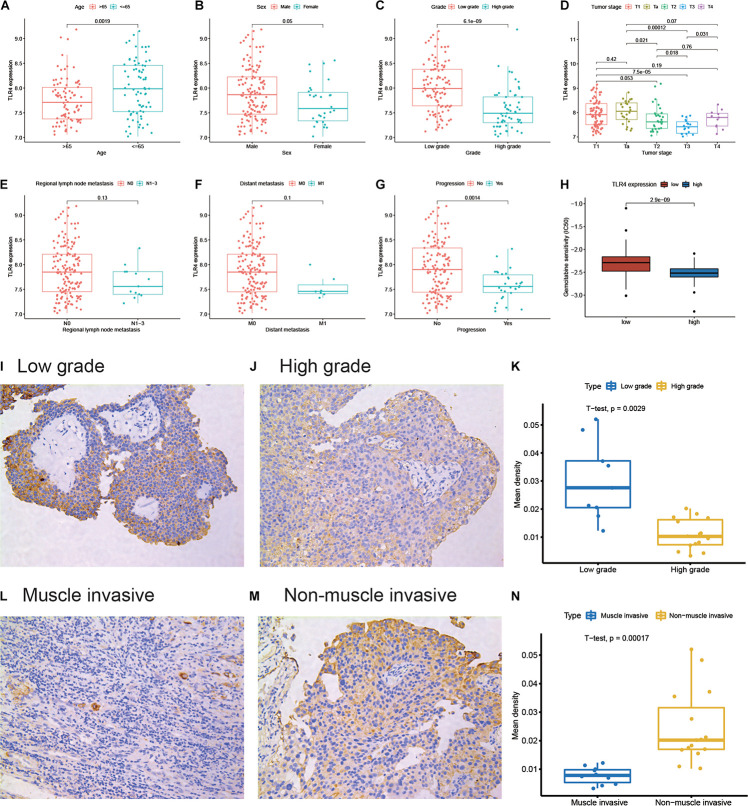
Differential expression of TLR4 in clinicopathological subgroups. The subgroups are performed for age **(A)**, sex **(B)**, pathological grade **(C)**, tumor stage **(D)**, regional lymph node metastasis **(E)**, distant metastasis **(F)**, and progression **(G)**. The correlation between TLR4 expression and gemcitabine sensitivity **(H)**. The representative TLR4 immunohistochemistry images for a low-grade sample **(I)** and a high-grade sample **(J)**. The quantitative result **(K)** is illustrated for a low-grade group (*n* = 9) and a high-group (*n* = 16). The representative TLR4 immunohistochemistry images for the muscle invasive sample **(L)** and non-muscle invasive sample **(M)**. The quantitative result **(N)** is illustrated for the muscle invasive group (*n* = 10) and non-muscle invasive group (*n* = 15).

To further explore the clinical implications of TLR4, we find that the expression of TLR4 is significantly lower in elderly patients (age >65 years), high pathological grade tumor, higher tumor stage, and progressive tumor (Figures 2A–D,G). However, these is no significant relationship between TLR4 and lymph node metastasis (*P* = 0.13) or distant metastasis (*P* = 0.1, Figures 2E,F). Gemcitabine and cisplatin are the standard systemic chemotherapy in MIBC patients. Patients in the low TLR4 expression group are sensitive to gemcitabine (*P* < 0.001, Figure 2H). But the cisplatin sensitivity is comparable between the high TLR4 expression and the low TLR4 expression groups (Supplementary Figure 1). The result can help to recognize chemotherapy-sensitive patients. We also apply IHC to validate the results. Twenty-five bladder cancer patients are enrolled in the cohort. Figures 2I,J show the representative images of low-grade and high-grade tissues, respectively. The patients with low-grade tumor in our cohort have higher expression of TLR4 than the patients with high-grade tumor (*P* = 0.0029, Figure 2K). Figures 2L,M show the representative images of MIBC and NMIBC, respectively. The patients with NMIBC have higher expression of TLR4 than the patients with MIBC (*P* = 0.0002, Figure 2N).

### Promoter Methylation Level of TLR4

**FIGURE 3:**
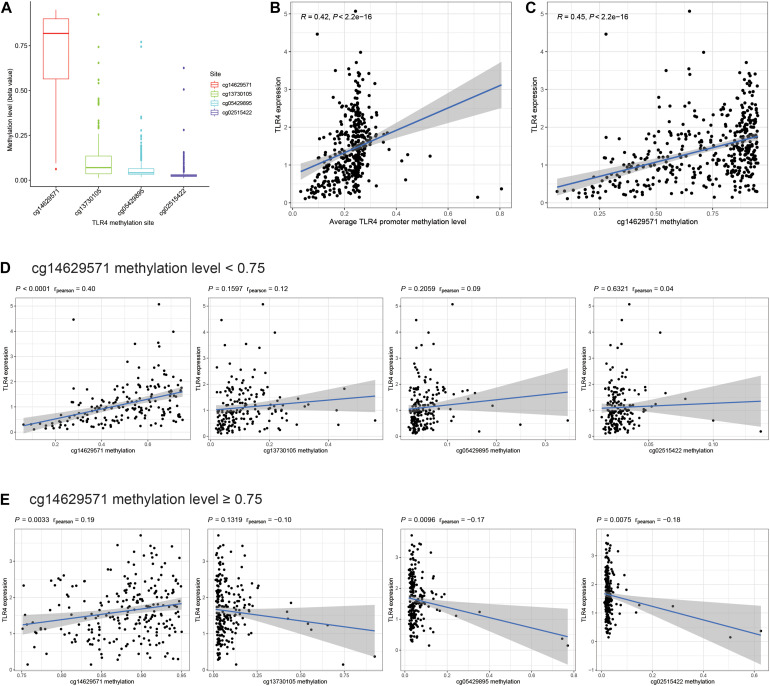
Comprehensive analysis of TLR4 promoter methylation sites. **(A)** The methylation levels (beta value) of four promoter methylation sites of TLR4 in TCGA. **(B)** Correlation between TLR4 expression and the average TLR4 promoter methylation level. **(C)** Correlation between TLR4 expression and the methylation level of cg14629571. **(D)** Correlation between TLR4 expression and the methylation levels of each site in patients with a cg14629571 methylation level less than 0.75. **(E)** Correlation between TLR4 expression and the methylation levels of each site in patients with a cg14629571 methylation level greater than or equal to 0.75.

Promoter methylation is an important mechanism accounting for the changes in gene expression. Therefore, we aim to explore the promoter methylation level of TLR4. There are four promoter methylation sites in TLR4: cg14629571, cg13730105, cg05429895, and cg02515422. The cg14629571 site has the highest methylation level (Figure 3A). The expression of TLR4 is significantly positively correlated with the average methylation level of the TLR4 promoter (Figure 3B). Among the four sites, cg14629571 (Figure 3C) rather than cg13730105, cg05429895, and cg02515422 (Supplementary Figure 2) is significantly positively correlated with TLR4 expression (*R* = 0.45, *P* < 0.001). The correlation plot of cg14629571 and TLR4 expression also indicates that a considerable number of samples have a high cg14629571 methylation level (beta value > 0.75). Therefore, we conducted a subgroup analysis. In the cg14629571 methylation level < 0.75 group (Figure 3D), only cg14629571 is significantly associated with TLR4 expression (*R* = 0.40, *P* < 0.0001). However, in the cg14629571 methylation level ≥ 0.75 group (Figure 3E), there are weak but significant associations between TLR4 expression and cg14629571 (*R* = 0.19, *P* = 0.0033), cg05429895 (*R* = −0.17, *P* = 0.0019), or cg02515422 (*R* = −0.18, *P* = 0.0075). The results reveal that there might be a multistage methylation mechanism to regulate TLR4 expression. Moreover, the low cg14629571 methylation level is also associated with higher stage, high pathological grade, and higher tumor stage but not for lymph node metastasis and distant metastasis (Supplementary Figure 3). However, the difference of the OS rates between the high cg14629571 methylation group and the low cg14629571 methylation group is not significant for all patients (*P* = 0.246), patients with the cg14629571 methylation level <0.75 (*P* = 0.241), or patients with the cg14629571 methylation level (*P* = 0.131; Supplementary Figure 4).

### CIBERSORT and Gene Set Enrichment Analysis

**FIGURE 4:**
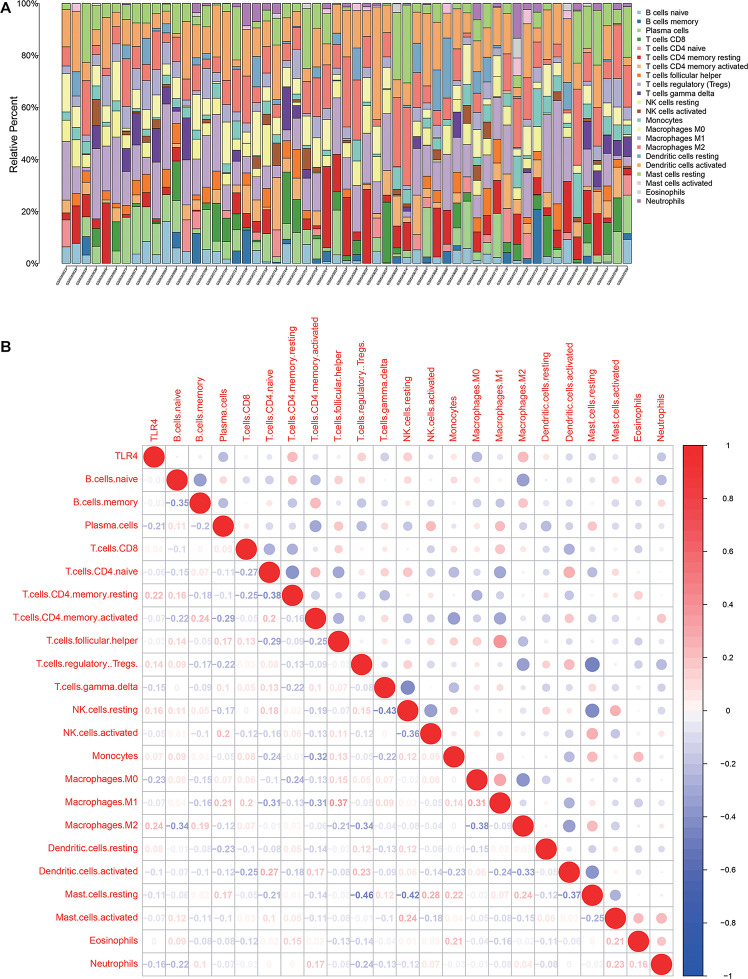
Tumor microenvironment of tumor tissues from GSE13507. **(A)** Infiltrating immune cell profiles of each sample with *P* < 0.05. **(B)** Correlation between TLR4 expression and infiltrating immune cells.

**FIGURE 5:**
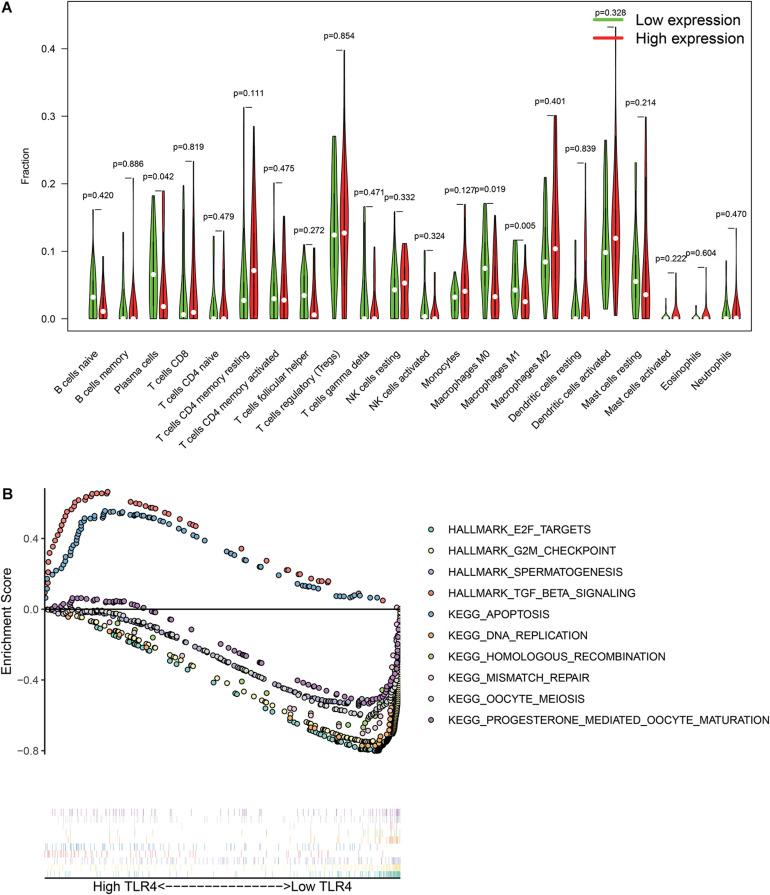
Differentially immune cell infiltration **(A)** and alterative pathways **(B)** between high TLR4 expression groups and low TLR4 expression groups.

There are 57 observations left after excluding samples with *P* ≥ 0.05 in CIBERSORT estimation (Figure 4A). The correlation between TLR4 and different types of immune cells is shown in Figure 4B. We compare the immune cells’ infiltration difference between the high TLR4 expression and low TLR4 expression groups (Figure 5A). Tumors with low TLR4 expression have significantly higher infiltrating levels of plasma cells, M0 Macrophages, and M1 Macrophages. CD8^+^ T cells play an indispensable role in an antitumor environment by exerting a cytotoxic effect and interacting with cytokines. We further investigate whether the change of TLR4 is correlated with these pathways (Supplementary Table 1). The interleukin-4 (IL-4), interferon-γ (IFN-γ), and IL-7 pathways are significantly up-regulated in tumors with a high level of TLR4. But the activation levels of the CTL pathway and T cytotoxic pathway are comparable between the high TLR4 expression and low TLR4 expression groups.

The GSEA checks the differentially activated pathways between the two groups and suggests that TGF-β signaling and apoptosis pathways are significantly enriched in the high TLR4 expression group. Moreover, the E2F targets, G2M checkpoint, spermatogenesis, DNA replication, homologous recombination, mismatch repair, oocyte meiosis, and progesterone-mediated oocyte maturation are significantly enriched in the low TLR4 expression group (Figure 5B).

## Discussion

In this research, we have shown that the expression of TLR4 is down-regulated during the initiation and development of bladder cancer. A low level of TLR4 can effectively forecast poor outcomes for bladder cancer patients. The result is consistent with a previous report (Kusuhara et al., 2019). Moreover, the expression of TLR4 is lower in the elderly, high grade tumors, higher tumor stage, and progressive tumors. Interestingly, a low TLR4 level is associated with higher gemcitabine sensitivity. These results emphasize the clinical implication of TLR4 to predict prognosis and drug response. Furthermore, we reveal the mechanism of expression alteration from the perspective of promoter methylation. The methylation level of cg14629571 site is positively related to TLR4 expression. TLR4 is an essential receptor in tumor microenvironment. We explore the relationship between TLR4 and infiltrating immune cells as well as related pathways. These outcomes provide an insight into the abnormal expression of TLR4 in bladder cancer.

The expression profile of the TLRs is assessed by comparing bladder cancer tissues, surrounding tumor tissues, and normal bladder tissues. Our findings reveal that the expression levels of TLRs are generally dysregulated in the surrounding tissues or tumor tissues of bladder cancer. These results are in accordance with other studies. Sabah-Ozcan et al. (2017) also detected significant decreases in the expression of TLR2-7 and TLR10 in bladder cancer but failed to observe changes in the expression of TLR1, TLR8, and TLR9. Ayari et al. (2011) systematically analyze TLR expression in FFPE human bladder cancer tissues. The results indicate that all normal urothelial samples have TLR2, TLR3, TLR4, TLR5, TLR7, and TLR9 staining. Most NMIBC samples show positive staining for these TLRs, but the intensity is significantly lower than in normal bladder samples. The expression of TLRs is further decreased in MIBC. Therefore, the family of TLRs participates in the development of bladder cancer and could be potential therapeutic targets.

In our research, TLR4, rather than other TLRs, shows prognostic value to predict OS and CSS in bladder cancer patients. This result leads to further exploration of TLR4. BCG as a TLR4 agonist is the first-line intravesical treatment for NMIBC. Ibarra et al. report that BCG can induce the synthesis and secretion of proinflammatory cytokines by activating TLR4 (Ibarra et al., 2019). The secretion of cytokines is affected by differentially expressed TLR4 (Olbert et al., 2015b). We speculate that TLR4 can be served as a favorable predictor of BCG responsiveness for NMIBC patients. Future studies are expected to establish their relationship. On the other hand, intriguingly, we find that the TLR4 expression level can predict drug sensitivity. Patients with low TLR4 levels are more sensitive to gemcitabine. Gemcitabine is the standard chemotherapy in MIBC patients, which indicates that the detection of TLR4 expression may help to recognize those patients who respond to systemic chemotherapy and optimize the dosage to improve the efficacy. In NMIBC patients, our previous network meta-analysis concluded that gemcitabine can be used as an intravesical instillation therapy with similar efficacy to BCG (Lu et al., 2020). A precision treatment strategy could be provided that patients with high TLR4 expression are recommended to receive BCG treatment due to improved immune response, while patients with low TLR4 expression are advised to receive gemcitabine treatment due to high sensitivity.

The toll-like receptor 4 is expressed not only in malignant cells but also in immune cells. Lipopolysaccharide (LPS), a TLR4 agonist, can induce anti-inflammatory cytokine secretion (e.g., IL-8) by activating TLR4 expressed in cancer cells (Moghadam and Nowroozi, 2019; McCall et al., 2020). LPS can also regulate TLR4 to induce IL-6 response through MAPK and PI3K pathways, which promote tumor progression (Qian et al., 2009). TLR4 expressed on CD4^+^ T cells can be activated to increase IFN-γ production after receiving the combined stimulation of maltose-binding protein and BCG (Liu et al., 2018). Astragalus polysaccharides, a kind of traditional Chinese medicine, can modulate macrophages through TLR4/MyD88 signaling to enhance the immune response in breast cancer patients (Zhou et al., 2017). On either tumor cells or immune cells, TLR4 is involved in the process of initiation and treatment of cancer. These reports also reveal that the activation of TLR4 plays both antitumor and pro-tumor roles. The opposite effects may result from different ligands of TLR4. LPS generally induces immunity tolerance in microphages, while BCG or β-glucan can boost innate immune memory by means of epigenetic regulation of histones (Saeed et al., 2014). Therefore, the TLR agonists discovered for cancer treatment should be carefully evaluated.

Promoter methylation may explain the differential gene expression pattern in tumors (Baylin, 2005). We report that TLR4 expression is positively related to TLR4 promoter methylation, especially the cg14629571 site. Nevertheless, the expression of TLR4 is correlated to cg14629571, cg05429895, and cg02515422 when the cg14629571 site is hypermethylated. It suggests that the expression of TLR4 may be controlled by a multistage mechanism. In the early stages of bladder cancer development, TLR4 is regulated by multiple methylation sites. High TLR4 levels maintain the immune response and anti-tumor microenvironment (Chen et al., 2018; Shetab et al., 2018). In the process of tumor progression, the cg14629571 site dominates the expression of TLR4. Further experiments could be designed to validate the methylation regulation of TLR4.

The tumors with high TLR4 expression have low infiltrated levels of plasma cells, M0 macrophages, and M1 macrophages with activated TGF-β and apoptosis pathways. High expression of Smad2 and Smad4 in the TGF-β signaling pathway predicts longer OS in bladder cancer patients, while TGF-β1 is inversely correlated with the survival rate (Stojnev et al., 2019). BCG can stimulate TLR2/4 in bladder tumor cells to decrease cell proliferation and up-regulate apoptosis (Camargo et al., 2018). The activation of TLR4 can also induce apoptosis in multiple cancer types, such as leukemia, liver cancer, and colon cancer (Ren et al., 2018; Xiao et al., 2018; Lee et al., 2020). Cytotoxic effects and cytokine production of immune cells exert antitumor effects in the immune microenvironment. We find that the increase of TLR4 is accompanied by the up-regulation of the IL-4 pathway, the IFN-γ pathway, and the IL-7 pathway. But the T cytotoxic pathway is unchanged between high TLR4 and low TLR4 tumors. The results can promote further research on the tumor environment reprogramming of bladder cancer.

## Conclusion

The study outlines the alterative expression and prognostic value of the TLR family in bladder cancer. TLR4 is a favorable prognostic gene to predict OS and CSS in bladder cancer patients. Diminished TLR4 expression is associated with the malignant behavior of tumors (high pathological grade, higher tumor stage, and progression). Low TLR4 can also forecast higher drug sensitivity to gemcitabine. The TLR4 promoter methylation level of the cg14629571 site can account for the reduced expression of TLR4. Tumor-infiltrated immune cell analysis and GSEA reveal the potential function of TLR4 in the initiation and development of bladder cancer.

## Data Availability Statement

The datasets presented in this study can be found in online repositories. The names of the repository/repositories and accession number(s) can be found in the article/Supplementary Material.

## Ethics Statement

The studies involving human participants were reviewed and approved by Medical Ethics Committee of Tongji Hospital Affiliated to Tongji Medical College of Huazhong University of Science and Technology Approval number: TJ-IRB20200729. Written informed consent for participation was not required for this study in accordance with the national legislation and the institutional requirements. Written informed consent was not obtained from the individual(s) for the publication of any potentially identifiable images or data included in this article.

## Author Contributions

J-LL and Q-DX: design, analysis and interpretation of data, drafting and critical revision of the manuscript, statistical analysis, and writing—original draft. J-LL, Q-DX, YS, YX, H-LH, C-QL, J-XS, J-ZX, JH, and S-GW: methodology and writing—review and editing. JH and S-GW: project administration. All authors contributed to the article and approved the submitted version.

## Conflict of Interest

The authors declare that the research was conducted in the absence of any commercial or financial relationships that could be construed as a potential conflict of interest.

## Footnotes

1 https://www.ncbi.nlm.nih.gov/geo/

2 http://xena.ucsc.edu/

3 https://www.gsea-msigdb.org/gsea/msigdb/index.jsp

## Abbreviations

TLR: Toll-like receptors
BCG: Bacille Calmette-Guérin
NMIBC: non-muscle invasive bladder cancer
MIBC: muscle invasive bladder cancer
GEO: Gene Expression Omnibus
TCGA: The Cancer Genome Atlas
OS: overall survival
CSS: cancer specific survival
IHC: immunohistochemistry
FFPE, formalin-fixed: paraffin-embedded
MOD: mean optical density
GSEA: gene set enrichment analysis
IL: interleukin
IFN: interferon.
