# Supplementary material for: Toll-Like Receptor 4 as a Favorable Prognostic Marker in Bladder Cancer: A Multi-Omics Analysis
Source: Front Cell Dev Biol. 2021 Jun 1;9:651560. doi: 10.3389/fcell.2021.651560 (PMC8204102; doi:10.3389/fcell.2021.651560)
Supplement: Supplementary file 1 [file Data_Sheet_1.docx]

Supplementary Materials

sTable 1: The differences in the pathways that related to cytokine and CD8^+^ T cell between TLR4 high and low groups.

sFigure 1: The relationship between TLR4 expression and cisplatin sensitivity (IC50).

sFigure 2: The correlation between TLR4 expression and cg13730105 (A), cg05429895 (B), and cg02515422 (C) methylation level.

sFigure 3: The association between cg14629571 and patient characteristics as well as tumor characteristics.

sFigure 4: The prognostic value of cg14629571 methylation level in bladder cancer.

sTable 1: The differences in the pathways that related to cytokine and CD8^+^ T cell between TLR4 high and low groups.

| Pathway Name | NES | NOM *p*-val | FDR *q*-val |
| --- | --- | --- | --- |
| BIOCARTA_IL4_PATHWAY | 1.613373 | 0.005941 | 0.375561 |
| BIOCARTA_IFNG_PATHWAY | 1.582141 | 0.011881 | 0.345493 |
| BIOCARTA_IL7_PATHWAY | 1.559785 | 0.034343 | 0.380599 |
| BIOCARTA_TGFB_PATHWAY | 1.45672 | 0.052314 | 0.483651 |
| BIOCARTA_IL17_PATHWAY | 1.357803 | 0.143141 | 0.524792 |
| BIOCARTA_IL1R_PATHWAY | 1.308749 | 0.143984 | 0.528883 |
| BIOCARTA_IFNA_PATHWAY | 1.251742 | 0.203187 | 0.575345 |
| BIOCARTA_CYTOKINE_PATHWAY | 1.2341 | 0.239351 | 0.589337 |
| BIOCARTA_IL2RB_PATHWAY | 1.175981 | 0.228963 | 0.588551 |
| BIOCARTA_CTL_PATHWAY | 0.967001 | 0.569106 | 0.708365 |
| BIOCARTA_IL12_PATHWAY | 0.928441 | 0.530121 | 0.710173 |
| BIOCARTA_IL3_PATHWAY | 0.882892 | 0.603586 | 0.764814 |
| BIOCARTA_CCR3_PATHWAY | 0.854174 | 0.64902 | 0.788111 |
| BIOCARTA_TCRA_PATHWAY | 0.848902 | 0.674897 | 0.787798 |
| BIOCARTA_TCYTOTOXIC_PATHWAY | 0.841705 | 0.70082 | 0.795854 |
| BIOCARTA_CCR5_PATHWAY | 0.841168 | 0.671518 | 0.792067 |
| BIOCARTA_IL2_PATHWAY | 0.800367 | 0.760649 | 0.820763 |
| BIOCARTA_IL10_PATHWAY | 0.775432 | 0.776423 | 0.833836 |
| BIOCARTA_IL5_PATHWAY | 0.759739 | 0.757829 | 0.823161 |
| BIOCARTA_IL6_PATHWAY | 0.667337 | 0.855159 | 0.918321 |
| BIOCARTA_CTLA4_PATHWAY | 0.618877 | 0.902692 | 0.9452 |


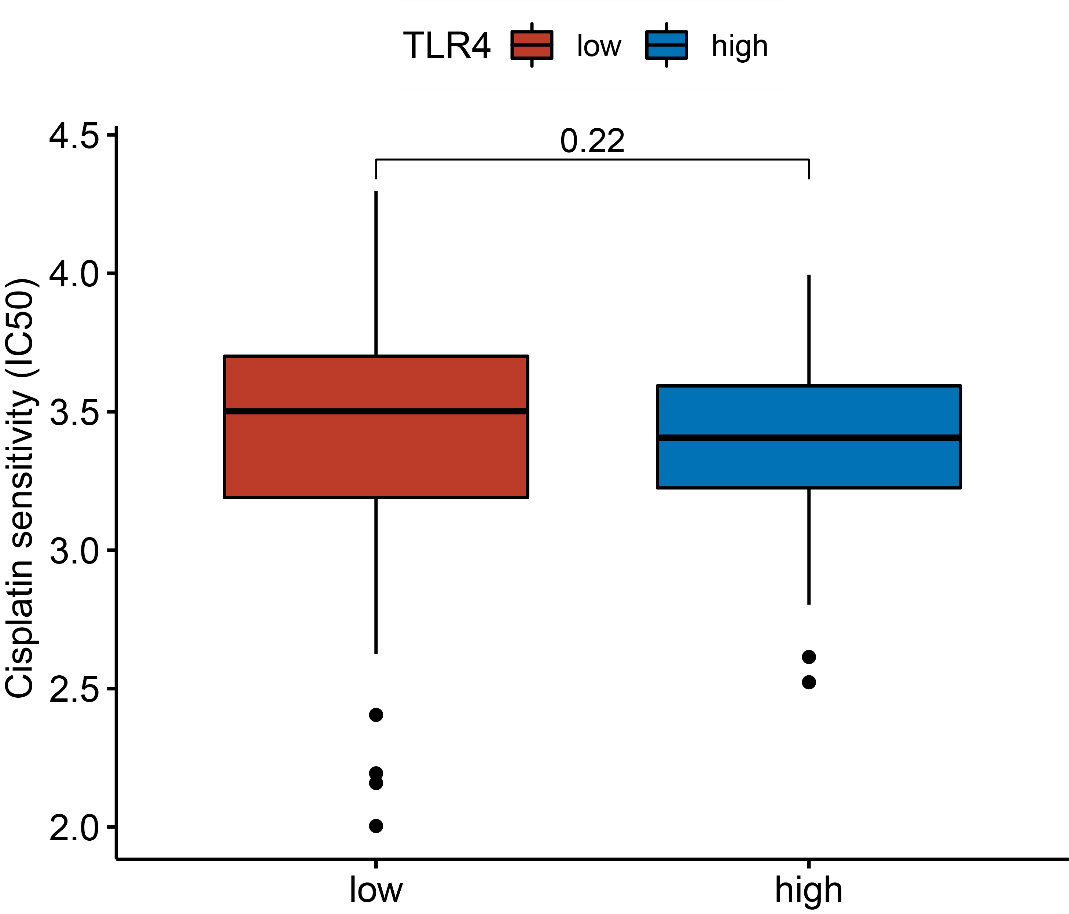
sFigure 1: The relationship between TLR4 expression and cisplatin sensitivity (IC50).

sFigure 2: The correlation between TLR4 expression and cg13730105 (A), cg05429895 (B), and cg02515422 (C) methylation level.


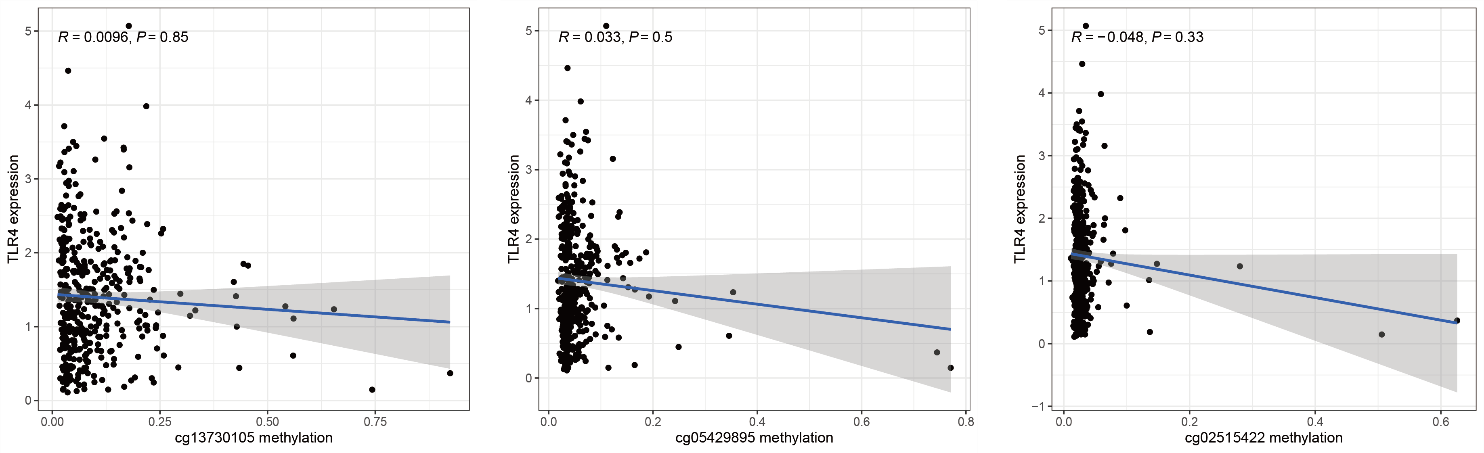


sFigure 3: The association between cg14629571 and patient characteristics as well as tumor characteristics.


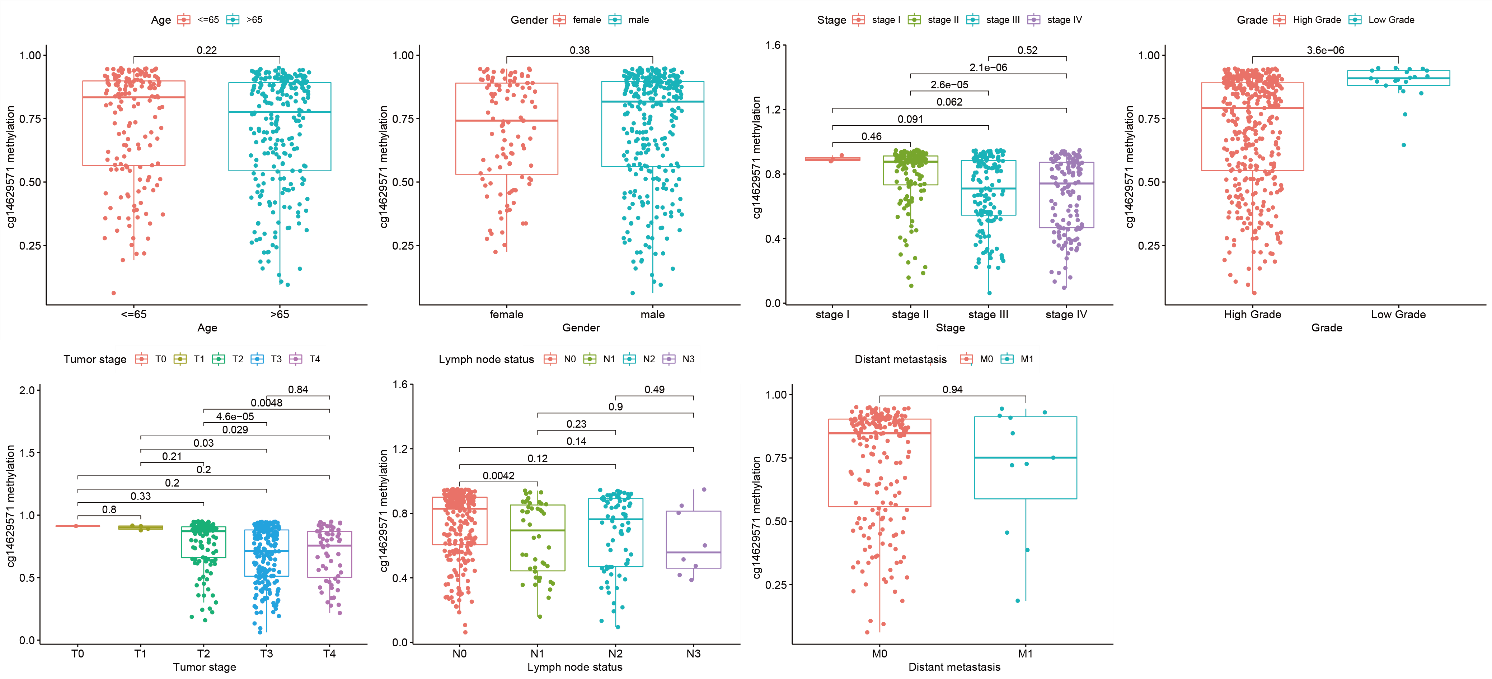


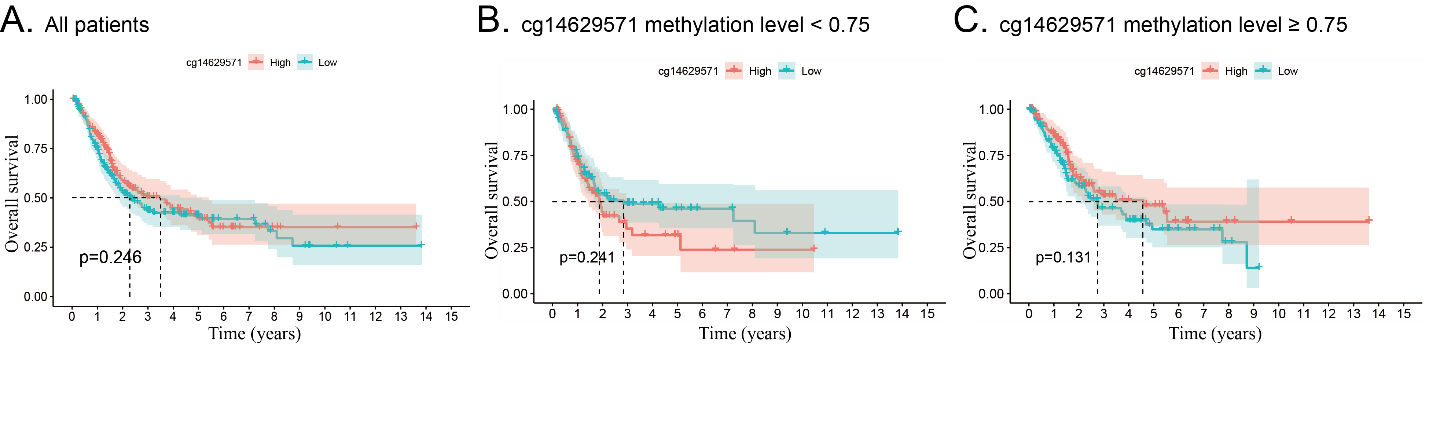
sFigure 4: The prognostic value of cg14629571 methylation level in bladder cancer.
